# Aging and the Combined effects of ADRA2B and CB1 deletions on Affective Working Memory

**DOI:** 10.1038/s41598-019-40108-5

**Published:** 2019-03-11

**Authors:** Beth Fairfield, Nicola Mammarella, Lara Fontanella, Annalina Sarra, Marco D’Aurora, Liborio Stuppia, Valentina Gatta

**Affiliations:** 1 Department of Psychological, Health and Territorial Sciences, “G. d’Annunzio” University, Chieti-Pescara, Via Dei Vestini 31, 66100 Chieti, Italy; 2 CeSI-Met, “G. d’Annunzio” University, Chieti-Pescara, Via Dei Vestini 31, 66100 Chieti, Italy; 3 Department of Economics, “G. d’Annunzio” University, Chieti-Pescara, Viale Pindaro 42, 65127 Pescara, Italy

## Abstract

Many studies have found that memory for affective material is better than memory for neutral information and memory for positive material compared to negative material is better in older adults. Behavioral, neurophysiological as well as single polymorphism differences have been advanced to account for these effects. Here, we aimed to examine whether the combination of two polymorphisms (ADRA2B and CB1) in older adults influences active maintenance and manipulation of emotional information in aging working memory. We examined genotype data from 207 older adults (56 double deletion carriers, 116 single deletion carriers and 35 no deletion carriers) who performed a verbal operation span-like task with positive, negative and neutral words. We found that subjects carrying both ADRA2B and CB1 variants generally remembered a higher number of words. In addition, double carriers showed positivity effects while single carriers showed more general emotional enhancement effects, especially as strings lengthened. These findings are amongst the first to suggest a haplotype account of positivity effects in older adults’ memory.

## Introduction

Older adults generally show poorer performance in working memory and especially in complex span tasks that require active maintenance of the to-be-remembered information^1^. Different explanations have been advanced to explain these declines and although working memory may be affected by more general cognitive abilities such as speed of processing^2^ declines in processing resources^3^ and/or inhibition^4^ other studies show how age-related changes in mechanisms responsible for maintaining to-be-remembered information may also be implicated^5,6^. Indeed, working memory impairments may also be linked to factors involved in the active maintenance of information in working memory.

In particular, Baddeley and Hitch^5^ posit that verbal traces are stored in the phonological loop through rehearsal, or a subvocal repetition of the to-be-remembered information. This is particularly interesting when testing older adults with span tasks where performance on short span tasks is generally better than performance on longer spans. Indeed, if information is maintained through rehearsal, short spans that require shorter articulation times, should be rehearsed more than long spans and subsequently lead to an increase in memory performance.

Differently, information may be maintained in working memory through an attention-based mechanism. In line with the Time-based Resource-sharing model^6^, attentive processes are involved and since limited, must be shared, in both the processing and maintenance of information in working memory. In particular, if attentive resources are employed for processing, they are not available for maintenance and vice versa. Importantly, in span tasks where attention is necessarily directed at one item at a time, memory traces for the other items decline as attention must be continuously shared between processing and maintenance.

Nonetheless, recent behavioral studies highlight how affective information processing in working memory is preserved in aging^7^ and many studies have highlighted how memory for affective information is maintained with age^8^ and in particular, how older adults often remember positive information better than negative information (positivity effect). Carstensen and Mikels^9^ posited that advantage for positive information is linked to affective goals that modify attentive processes in older adults. That is, older adults who prefer affective information, direct their attentive resources to this information and subsequently show an increase in memory for affective information in general, and for positive information, in particular (but see^10,11^ for different results).

Neurophysiological explanations suggest that older adults remember affective information better than neutral information because the ventromedial prefrontal cortex (PFC), the anterior cingulate gyrus, and the temporal pole, involved in affective information processing^11,12^, are less affected by aging than other regions involved in general cognitive processing. In addition, older adults generally show an increase in amygdala activity in response to positive information and a decrease in amygdala activity in response to negative information^13,14^.

Another possible explanation for affective working memory performance in older adults may be linked to genotype differences^15^. In particular, ADRA2B, a functional deletion in the adrenoceptor gene 2B, and CB1, a cannabinoid receptor type *1*, have been separately linked to emotional memory performance^16–18^. To our knowledge however, no study has investigated the combined action of these genotype differences on an affective working memory task in older adults.

## The role of ADRA2B and CB1 receptor in affective processing

Studies on the interaction between ADRA2B variant and emotion, with a specific focus on valence effects, show a very complex picture (for a review see^19^). Behavioral studies have that found a bias for negative information^20,21^, an association with suicidal behavior^22^ and a decrease in false memories^23^ in ADRA2B carriers. Others have detected more general emotion effects since ADRA2B carriers were sensitive to both positive and negative stimuli^19,24–27^. In particular, Mammarella *et al*.^28^ suggested that ADRA2B may also mediate traumatic memories in older adults since ADRA2B carriers remembered positive words better than negative and neutral words even when these were pronounce with a negative prosody (see Mammarella *et al*.^28^ for a detailed description). fMRI data also shows an increase in the neural activity in the amygdala while encoding affectively connoted images in older adults and especially in ADRA2B carriers^20^ and several studies^28^ have underlined how the increase in the noradrenergic system generally found in older adults may reflect a persistent focus on affective information in these individuals, especially in ADRA2B carriers. Finally, since the noradrenergic system may also be, in part, responsible for cognitive-affective flexibility and cognitive reserve^29^, and therefore crucial for general emotion regulation processes in aging, ADRA2B carriers may benefit even more^28^.

The intronless gene ADRA2B is located on the 2p13-q13 chromosome and encodes a seven-pass transmembrane protein widely distributed in the human central and peripheral nervous systems. The presence of the ADRA2B functional polymorphism, consisting of inframe three glutamic acids residues deletion (301–303) in the third intracellular loop, reduces receptor functionality and increases central noradrenergic transmission, through the presynaptic inhibition of NA release.

Recent studies have also begun investigating the role that the endocannabinoid system may play on cognitive-affective flexibility that depends on WM functions including goal maintenance, inhibition, shifting and updating^18,30,31^. The endocannabinoid system is also associated with stress release and CB1 receptors in the prefrontal cortex may modulate anxiety, chronic stress, motivation, and, more generally, social functioning^30^. For example, CB_1_ receptors are essential for the extinction of conditioned fear associations, indicating an important role for this receptor in neuronal emotional learning and memory as shown by fMRI studies investigating the cognitive control processes regulating fear extinction and, in particular, on the role of prefrontal cortex^32^.

Studies on the endocannabinoid system^18,33–36^ focused on the rs2180619 single-nucleotide cannabinoid receptor type 1 polymorphism (CB1 receptor) located in the q14-ql5 region of chromosome 6. This receptor regulates the attenuation of synaptic transmission and psycho-activity by interfering with the release of other neurotransmitters. In this manner, CB1 protects the central nervous system from overstimulation or inhibition by other neurotransmitters.

Considering the established role of the noradrenergic and cannabinoid systems in affective processing mentioned above, our study aimed to investigate the combined effect of ADRA2B and CB1 carriers on affective working memory performance in older adults. Indeed, the combination of multiple polymorphisms that interact in the same pathway may amplify the effect of the single variants. In general, we expect double carriers to show better memory performance than single and/or no variant carriers due to cognitive flexibility and cognitive resources. Finally, in line with the large corpus of data available in literature regarding positivity effects in older adults, double carriers may show a positivity effect and remember more positive words than negative and/or neutral ones.

## Results

**Figure 1:**
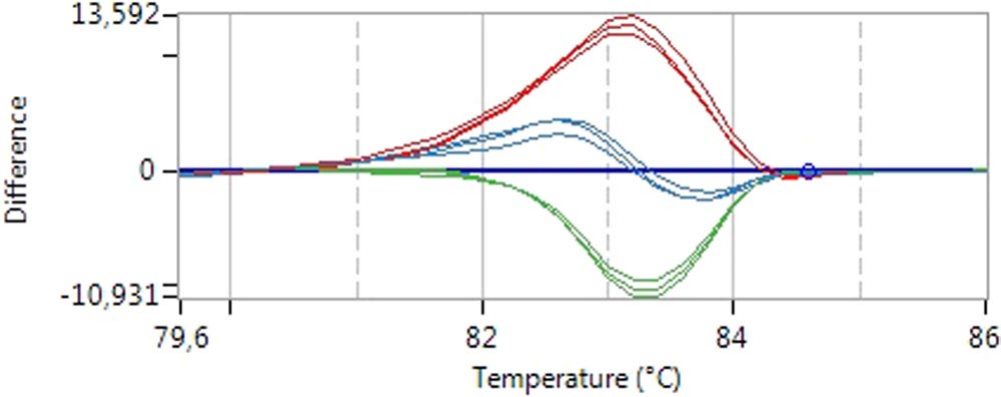
HRM analysis. The figure depicts an example of the HRM analysis of CB1 variants. The melting temperature of the amplicons carrying the different combination of alleles differs between homozygous and heterozygous subjects. In blue the profile of normal homozygous subjects, in red the profile of homozygous variant carriers and in green the profile of heterozygous subjects.

The ADRA2b polymorphism analysis identified 80 carriers and 127 non-carriers. The HRM analysis identified 57 homozygous carriers of the CB1 variant allele (GG; 27.54%), 91 heterozygous subjects (AG; 43.96%), and 59 homozygote subjects for the reference allele (AA; 28.50%) (Fig. 1). Genotype frequencies were in Hardy-Weinberg equilibrium (χ^2^ test P value > 0.05).

### Valence effects on the affective O-Span working memory task: an ANOVA

To investigate the specific valence effects on working memory in combined ADRA2B + CB1 carriers compared to single ADRA2B or CB1 carriers and non-carriers, we carried out a 4 (Genotype: ADRA2B carrier vs CB1 carrier vs ADRA2B + CB1 carrier vs None) X 3 (Valence: positive, negative, neutral) X 2 (Length: short, long) mixed ANOVA on accuracy scores as the dependent measure. Valence and length were within participant factors. We introduced the length of the WM task in the analysis since WM memory performance generally decreases as length increases and valence effects may emerge more clearly as available cognitive resources necessary for completing the task decrease.

The analysis revealed a significant genotype effect, F(2, 169) = 5,790, p < 0.01, ɳ_*p*_^*2*^ = 0.06. A Tukey-Kramer test for unequal sample size confirmed that carriers of both variants outperformed carriers of a single variant (ADRA2B or CB1). ADRA2B or CB1 single variant carriers showed comparable performance. The main effect of Valence was also significant, F(2,338) = 38,883, p < 0.001, ɳ_*p*_^*2*^ = 0.19. A post-hoc Tukey-Kramer test for unequal sample size confirmed that participants, in general, remembered positive words better than negative ones and negative words better than neutral ones, indicating classical positivity and emotional enhancement effects. A significant length effect was found F(1, 169) = 539,80, p < 0.001, ɳ_*p*_^*2*^ = 0.76 since performance was better on short strings than on longer strings. The 2-way interaction between genotype and length was significant, F(2, 169) = 11,590, p < 0.001, ɳ_*p*_^*2*^ = 0.12. A post-hoc Tukey-Kramer confirmed that performance was in all three groups decreased as strings lengthened and that carriers of both variants remembered more words than carriers of the single CB1 variant (p < 0.001) and carriers of the single ADRA2B variant (p < 0.05).

**Figure 2:**
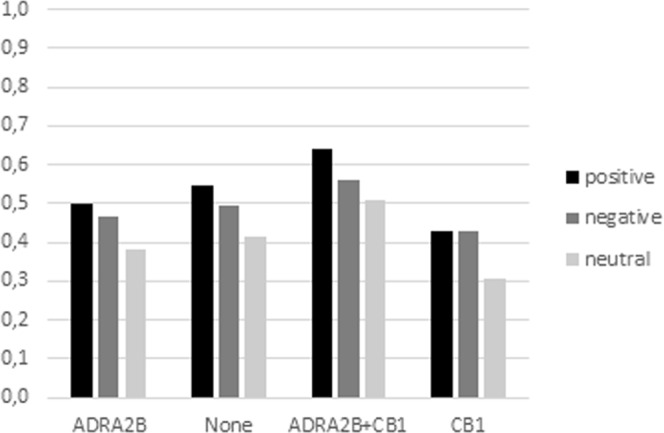
Total number of words remembered for long strings by genotype and valence.

Finally, a significant 3-way interaction between genotype, valence and length, F(6, 406) = 2,506, p = 0.02, ɳ _*p*_^*2*^ = 0.04 was found. A Tukey-Kramer test for unequal sample size confirmed that carriers (ADRA2B, CB1, ADRA2B + CB1) and non-carriers showed comparable performance on short strings. Instead, performance differed on longer strings. In particular, ADRA2b + CB1 carriers remembered positive words better than negative ones and negative words better than neutral ones whereas single variant carriers (ADRA2B or CB1) showed a more general preference for emotional words compared to neutral ones (Fig. 2).

### Cluster profiles of valence effects: a BPR approach

**Figure 3:**
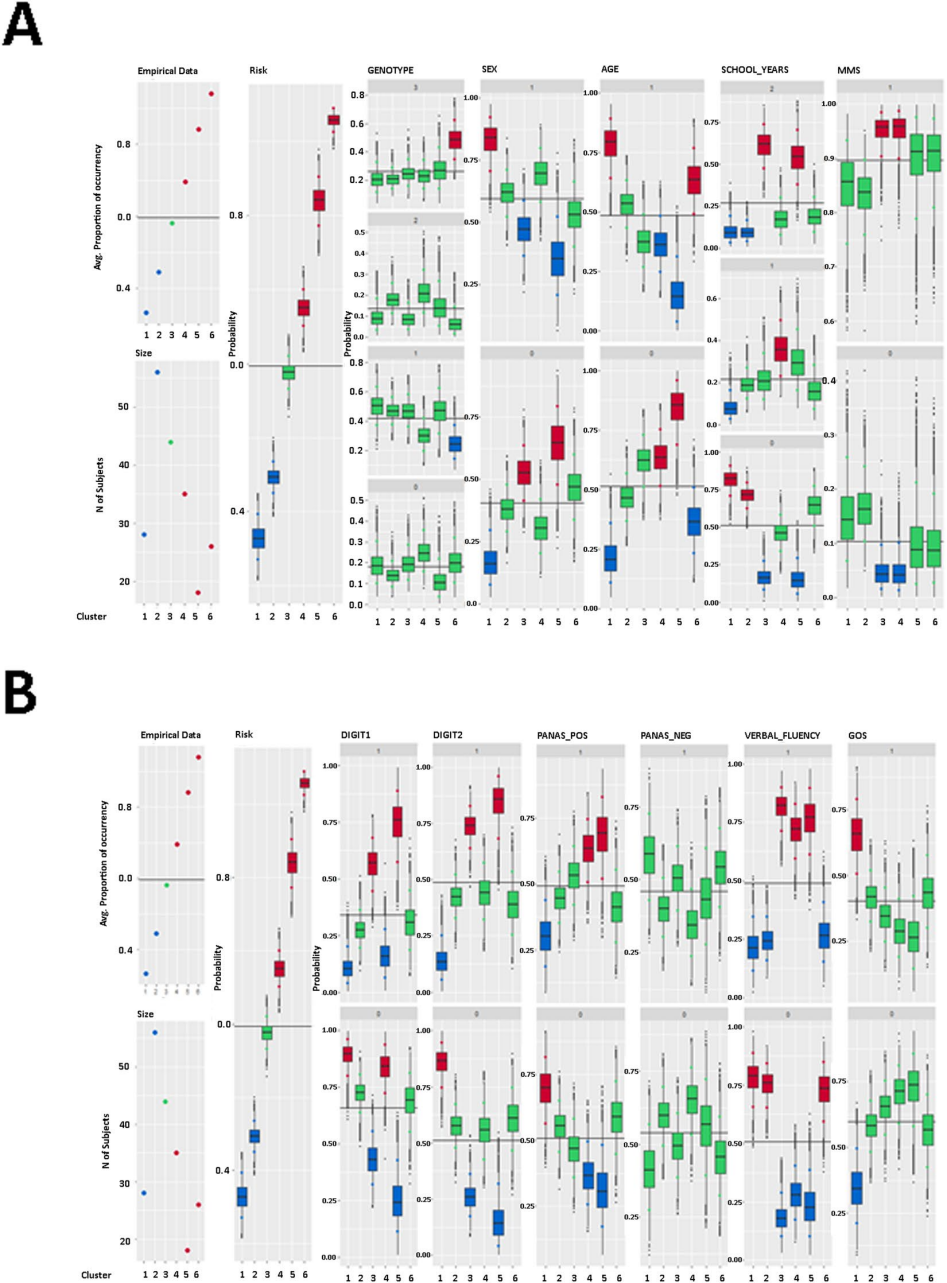
Total number of words remembered as response variable: summary plot of the posterior distribution of parameter ***ϕ***_*c*_, for *c* = 1, …, 6. Panel A shows the posterior distributions of the probability that a chosen covariate is equal to a given discrete category, across the identified clusters. In the coding of the ordinal categories, 0 corresponds to the lowest category and 1 to the highest. Finally, the box-plots are coloured according to the significance of the posterior estimates. red-coloured boxes indicate a performance above the average, green-coloured boxes indicate average performance, and blue-coloured boxes indicate a performance below average.

The total number of words remembered, regardless of valence, was considered as the outcome variable. The estimation procedure produced a partition of the joint distribution of affective O-span performance and related covariates into six clusters, composed of individuals with similar covariates and affective O-span performance. As a result, it was possible to evaluate the typical profile of participants for each identified group (Table 1). Clusters were sorted according to increasing performance: subjects belonging to the last three clusters showed an estimated performance significantly higher than the average, while subjects classified in the first two groups were less performant. The posterior distribution of all cluster specific parameters are represented in Fig. 3.

**Table 1:** Number of participants, performance means and 90% credible intervals for identified clusters and the whole sample.

Identifier	Cluster						Total
	1	2	3	4	5	6	
size	28	56	44	35	18	26	207
percentage	13.5	27.1	21.3	16.9	8.7	12.6	100
performance	0.36*	0.45*	0.59	0.68*	0.82*	0.93*	0.60
*90% CI*	*(0*.*34*, *0*.*40)*	*(0*.*43*, *0*.*47)*	*(0*.*57*, *0*.*61)*	*(0*.*65*, *0*.*70)*	*(0*.*79*, *0*.*85)*	*(0*.*91*, *0*.*95)*	*(0*.*59*, *0*.*61)*

(*) Provides information on coefficients significant at 90%.

To highlight specific features of the identified clusters, the estimated probability for each covariate was summarized to be equal to the different categories in the groups associated with lower (clusters 1, and 2) and higher levels of performance (clusters 4, 5 and 6) on the O-span test (Table 2).

**Table 2:** Posterior estimates of the probability for each categories in the clusters associated with lower (1 and 2) and higher (4, 5, and 6) levels of performance on the O-span test and average over all the clusters.

Covariates	Category	Cluster					Total
		1	2	4	5	6	
GENOTYPE	Adra2b = 0 e CB1 = 0 (0)	0.19	0.14	0.25	0.11	0.20	0.18
	Adra2b = 0 e CB1 = 1(1)	0.50	0.47	0.30	0.47	0.25*	0.42
	Adra2b = 1 e CB1 = 0 (2)	0.09	0.18	0.21	0.14	0.07	0.14
	Adra2b = 1 e CB1 = 1 (3)	0.21	0.21	0.24	0.28	0.49**	0.26
SEX	M = 0	0.17**	0.38	0.30	0.64*	0.47	0.40
	F = 1	0.83**	0.62	0.70	0.36*	0.53	0.60
AGE	63–71 (0)	0.22**	0.47	0.64°	0.84**	0.37°	0.51
	71–93 (1)	0.78**	0.53	0.36°	0.16**	0.63°	0.49
Education (years)	0–5 (0)	0.82**	0.71**	0.46	0.16**	0.64	0.51
	5–8(1)	0.08*	0.19	0.36°	0.30	0.17	0.22
	8–20 (2)	0.10**	0.10**	0.18	0.54**	0.19	0.27
MMSE	21–24 (0)	0.15	0.17	0.05°	0.10	0.09	0.10
	24–30 (1)	0.85	0.83	0.95°	0.90	0.91	0.90
FORWARD DIGIT	0–6 (0)	0.89**	0.72	0.84*	0.25**	0.69	0.66
	6–13 (1)	0.11**	0.28	0.16*	0.75**	0.31	0.34
BACKWARD DIGIT	0–4 (0)	0.86**	0.57	0.56	0.15**	0.61	0.51
	4–12 (1)	0.14**	0.43	0.44	0.85**	0.39	0.49
PANAS_POS	0–33 (0)	0.69*	0.55	0.37°	0.31°	0.59	0.51
	33–86 (1)	0.31*	0.45	0.63°	0.69°	0.41	0.49
PANAS_NEG	0–18 (0)	0.42	0.60	0.65	0.56	0.46	0.54
	18–47 (1)	0.58	0.40	0.35	0.44	0.54	0.46
VERBAL_FLUENCY	0–7.6 (0)	0.78**	0.75**	0.28**	0.23**	0.73*	0.51
	7.6–21.3 (1)	0.22**	0.25**	0.72**	0.77**	0.27*	0.49
GDS	0–2 (0)	0.35**	0.58	0.71	0.73	0.57	0.60
	2–19 (1)	0.65**	0.42	0.29	0.27	0.43	0.40

Significance levels: 1% (**); 5% (*); 90% (°).

### High vs. low WM resources

In the typical profile of the cluster associated with the highest WM performance (cluster 6), shows a prevalence of subjects carrying both variants (ADRA2B and CB1 genes), whereas CB1 variant carriers are underrepresented. Moreover, participants belonging to this cluster are more likely to be between 71–93 years of age and have lower verbal fluency scores. Results in Table 3 suggest that group 5 is more likely to be composed of males, with a high level of education and aged between 63–71 years. In this group, variables linked to forward and backward digit span scores as well as to positive affective scale attainments and verbal fluency results are significant, showing how these participants scored higher on these variables. The majority of subjects in cluster 4 are between the ages of 63 and 71 and have a low-medium level of education (5–8 schooling years). Group 4 includes a prevalence of subjects performing higher on the Mini-Mental State Exam (MMSE), on the positive affective scale and on the verbal fluency test.

**Table 3:** Neuropsychological and demographic characteristics by genotype.

Test variable	ADRA2B (24)		CB1 (113)		DOUBLE (56)		NONE (35)	
	M	SD	M	SD	M	SD	M	SD
Age (in years)	72.8	7.6	73.9	7.2	71.9	7.4	71.0	6.6
Education (in years)	6.3	2.5	8.4	4.7	7.4	4.0	7.9	4.8
Forward Digit span	4.7	1.5	6.1	2.2	5.8	2.0	5.3	2.1
Backward Digit span	4.8	1.5	4.9	2.2	4.6	1.9	4.3	1.8
FAS	8.6	3.7	8.3	3.9	7.7	3.4	8.9	4.4
PANAS pos	33.5	6.9	32.6	7.8	34.3	11.3	33.7	7.4
PANAS neg	18.0	5.1	19.0	6.8	20.1	7.6	19.0	7.5

Different results characterize the first two clusters, associated with a lower WM capacity. The lowest performing cluster (group 1) is mainly composed of females, aged between 71–93 years, with lower levels of education. They also record low scores on the Forward and Backward Digit, positive PANAS and verbal fluency. Conversely, these subjects score higher on the Geriatric Depression Scale (GDS). As for group 2, the only significant variables were education and verbal fluency. No other covariates were significant.

### Valence processing in WM

**Figure 4:**
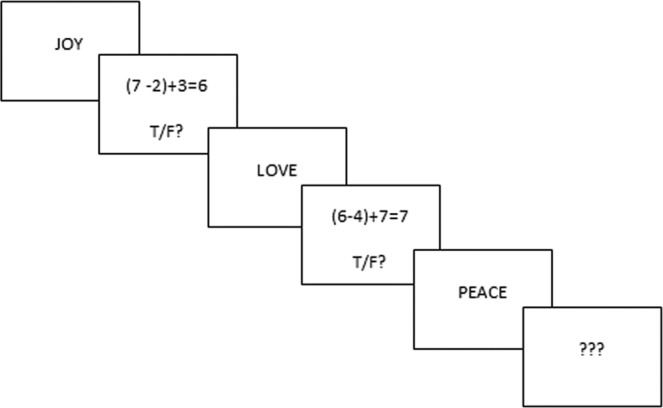
Graphical representation of a three-item set of the O-span.

In particular, this study aimed at investigating the role of genetic variability and the neuropsychological and demographic characteristics on affective O-span test performance, differentiated according to valence. The analyses including the number of positive, negative and neutral words remembered as response variable confirmed that enhanced WM performance was associated with a prevalence of subjects carrying both variants (ADRA2B and CB1). The empirical percentages of affective O-span performance across clusters, for different outcomes, are shown in the box-plots of Fig. 4. Results confirm that clusters 5 and 6 include individuals with enhanced memory performance. In particular, participants in clusters 5 and 6 remembered a higher percentage of positive words (Supplementary Fig. 1). In addition, participants in cluster 5 remembered a higher number of negative words than participants in other clusters (Supplementary Fig. 2). It must be noted, however, that the analyses for negative words revealed five clusters. Accordingly, cluster 5 for negative words corresponds to the enhanced WM performance and prevalence of both ADRA2B and CB1 carriers. Finally, the monochrome heatmap visualization (Supplementary Fig. 3) confirmed that carriers of both ADRA2b and CB1 deletion variants remembered more words, especially positive and negative ones, compared to single polymorphism carriers or non-carriers.

## Discussion

The present study aimed at investigating whether carriers of both ADRA2B and CB1 polymorphisms show different performance on an affective WM task in older adults compared to single variant carriers and no carriers. Moreover, for the first time, an attempt to establish a genetic and cognitive profile of older adults during affective processing was delineated. Results may be summarized as follows.

Regarding the influence of the combined effect of ADRA2B and CB1 compared to ADRA2B, CB1 or no variant on memory in an affective working memory task, we found that ADRA2B + CB1 carriers outperformed single variant carriers and no variant carriers with longer strings. That is, double carriers may have more cognitive resources and are able to use them to direct attentive resources towards motivated positive information during the processing and maintenance of information in the working memory task. Indeed, we found, a positivity bias in affective WM in carriers of both ADRA2B and CB1 polymorphisms whereas single variant carriers showed more general emotional enhancement effects since they remembered both positive and negative words better than neutral ones.

Working memory performance requires both efficient sub vocal rehearsal processes and the ability to share cognitive attentive resources between processing and maintenance requests. Older adults generally show good performance on simple span tasks that principally require rehearsal mechanisms for remembering. On the contrary, performance declines when cognitive resources must be shared. However, working memory performance, even on complex span tasks, increases in older adults when the task contains affective information. Moreover, in our study, older double carriers showed a preference for positive information during the affective WM task, suggesting that WM may function according to motivational goals (e.g., regulatory mechanisms) that direct attentive cognitive resources to the processing and maintenance of positive information and that this may be enhanced by the genetic profile.

The BPR analysis identified two groups of subjects with a prevalence of carriers of both analysed variants (ADRA2B and CB1), cluster 5 and 6 respectively, associated with enhanced WM performance. Although the older adults included in those clusters did not always show a stable cognitive baseline (lower verbal fluency in group 6), both groups showed higher WM performance. Interestingly, those clusters showed better memory for positive words (especially group 6). This emerging profile indicated that WM resources are crucial for focusing on positive information and that genotype-related differences may contribute to this valence bias.

In conclusion, our study confirmed a significant relationship between the noradrenergic system and memory for affective information in healthy older adults. Results confirm that affective information processing requires cognitive control mechanisms that selectively attend and remember positive information^8^. Moreover, this process may be modulated by ADRA2B and CB1 haplotype. Mather^8^ showed that NA influences stimulus processing priority and that priority becomes biased towards affective, and in particular, positive information during aging.

Our study also highlighted, for the first time, specific cognitive and genetic profiles. In particular, we found a high prevalence of both variant carriers in healthy aging^37,38^. Our study also confirms recent studies^29,38^ that show that both the noradrenaline and cannabinoid systems are crucial in affective information processes, and in positivity effects in memory in particular, in the aging brain^5–12^ (Mather and Harley^29^ Robertson^38^). Moreover, this may be also linked to the haplotypes of ADRA2B and CB1. The present data represent a novel starting point for evaluating how genetic differences may modulate affective working memory performance in older adults as well as in the healthy and pathological aging brain.

## Methods

### Ethics statement

The Departmental Ethics Committee at the University of Chieti approved the study. In accordance with the Declaration of Helsinki, all participants gave written informed consent prior to inclusion in the study.

### Participants

Sample size was established following the typical effect size (η^2^) of 0.07 of genetic correlates of memory studies^13^. We required 80 participants per genotype for a behavioral study. In line with the finding that 30% of the White population show the ADRA2B deletion variant, we recruited 221 right-handed native Italian speakers from a group of healthy community dwelling older adults from the Chieti area to participate in the study. Participants were between the ages of 64 and 91 (mean age 72.7; SD 7.2). Exclusion criteria included a history of significant head injuries, stroke, epilepsy and learning disabilities. Eight participants were excluded because they could not be genotyped for ADRA2B or CB1. A further 6 participants with previous or current diagnosis or treatment for anxiety and depression, revealed by a self-report demographics questionnaire were excluded leaving a total of 207 participants.

Neuropsychological and demographic data are reported in Table 3. All participants had normal or corrected-to-normal vision. All participants completed the forward and backward digit spans of the Wechsler Adult Intelligence Scale-Revised (WAIS-R^39^) and the Positive and Negative Affective Scale (PANAS^40^) to assess current mood.

### Affective Operation Span

**Figure 5:**
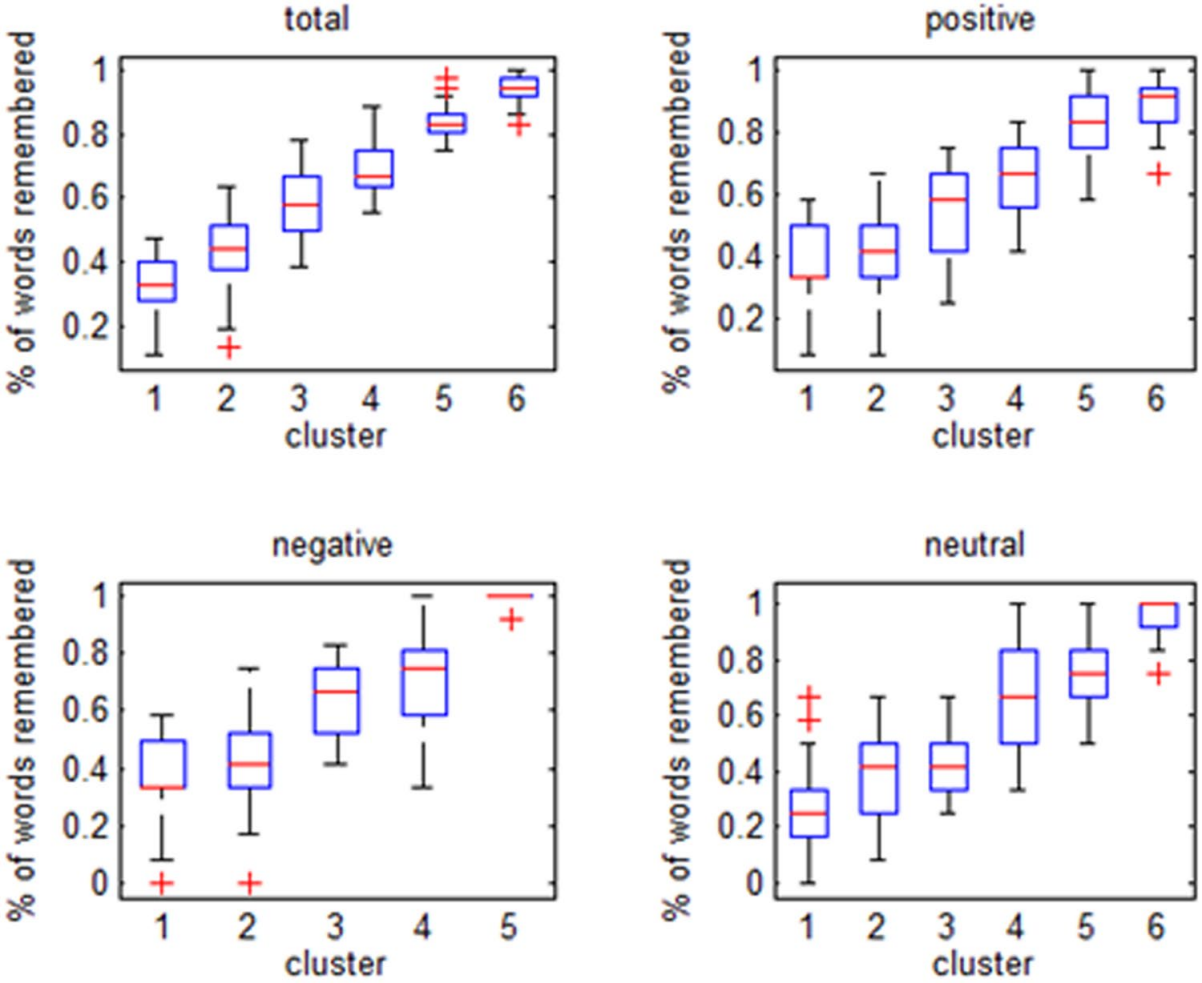
Affective O-span performance: box-plot of empirical percentages across clusters for different outcome variables.

A modified version of the Operation Working Memory Span (O-span) Test for emotional words (adapted from Turner & Engle^39^) was used. The O-span requires participants to solve a series of math equations while remembering series of semantically unrelated words. For each trial, an equation/word string appeared at the center of a computer monitor. Participants read the equation aloud, determined whether the given solution was true or false and then read the following word. The task was self-paced and participants pressed the space bar after reading the word in order to view the next equation–word string. The equation–word strings were presented in sets of three to six items. Three question marks in the center of the monitor cued the end of each set and to recall the words in the order of presentation (see Fig. 5 for a graphical representation of the O-span).

We constructed three to six equation-word strings for each affective valence (positive, negative and neutral) with two trials for each set size. The order of set size varied randomly. An 85% accuracy criterion on the math equations was required in order to ensure that participants were not trading off between solving the equations and remembering the words. The experimenter transcribed accuracy for math equations on a dedicated protocol. Two training trials preceded the experimental task. Thirty-six math equations and 36 target words^40^ were used for the operation span task. Positive words had a mean valence of 7.8 (1.5) and a mean arousal level of 5.9 (2.8), negative words had a mean valence of 2.4 (1.8) and a mean arousal level of 5.9 (2.7) and neutral words had a mean valence of 5.5 (1.9) and a mean arousal level of 2.7 (2.3). Valence set order was counterbalanced across participants. For a detailed description and psychometric properties of the O-span task, see previous studies^37,41^. The proportion of correctly words recalled regardless of serial position was considered as the WM measure.

### Genetic Analysis

Genomic DNA was isolated from buccal swabs using the NucleoSpin Tissue kit (Macherey-Nagel, Düren, Germany) according to manufacture instructions. DNA quality and quantity were assessed by Qubit 2.0 (ThermoFisher Scientific, Waltham, MA, USA).

### ADRA2B

PCR reactions for the polymorphisms analysis were carried out as previously reported^23^. In line with previous studies^21,26^ homozygote and heterozygote ADRA2B deletion carriers were treated as a single group due to the low number of homozygotes. A group of 207 participants was available for analysis. Homozygosity for the Glu301-Glu 303 deletion was detected in 4 participants; 76 participants were heterozygote while 127 were homozygote reference.

### CB1

Genotypes for the rs2180619 (*A* > *G*) were determined by High Resolution Melting (HRM) analysis^18^ (Fig. 3). Each sample was run in triplicate. According to the Ensembl database (http://www.ensembl.org/Homo_sapiens/Variation/Population?db=core;r=6:88167733-88168733;v=rs2180619;vdb=variation;vf=1566333), the distribution of our sample (G: 49%; A: 51%) the allelic distribution observed here is similar to European (G: 43.6%; A: 56.4%) and American (G: 49.6%; A: 50.4%) populations.

Following genotyping, participants were grouped as follows: 24 (11.6%) presented only the ADRA2B variant, 92 (44.4%) presented only the CB1 variant, 92 (27.1%) presented both ADRA2B and CB1 variants, 35 (16.9%) presented no variants. Groups matched for age and education, general short-term memory (measured with the forward and backward digit spans of the Wechsler Adult Intelligence Scale-Revised (WAIS-R^42^), verbal fluency and current mood (measured with the Positive and Negative Affective Scale, PANAS^43^). All p ≥ 0.995.

### Data Analysis

First, to clarify specific affective working memory performance between subjects carrying one variant (ADRA2B or CB1), both (ADRA2B + CB1) or none, we ran a mixed-model analyses of variance (ANOVAs) on affective O-span data (old words remembered regardless of order) with Valence (positive, negative and neutral) and span length (short and long) as repeated measures factor, and Genotype (ADRA2B vs CB1 vs. ADRA2B vs none) as the between-subjects factor.

Second, we ran a Bayesian Profile Regression to identify high and low performance clusters of individuals and to test the influence of ADRA2B or CB1 variants, isolated or in combination, on Affective Working Memory in older adults. We also investigated whether these clusters showed particular behavioural profiles.

### Bayesian Profile Regression

To examine the genotype-related effects on affective WM, we adopted a statistical technique known as Bayesian Profile Regression (BPR)^44^ and carried out statistical analyses with the freely available package PReMiuM^42^ for R statistical software (R Core Development Team 2018). This method allows us to explore the link between a response variable and a set of associated covariate data through cluster membership, so that the outcome and the clusters mutually inform each other^45^. It also allows us to include different neuropsychological and demographic characteristics potentially involved in the processing and maintenance of affective information in the O-span test variables in the analysis, in addition to those related to genetic variability.

The BPR assigns participants to clusters, based on the similarity of their covariates, and then associates clusters to affective O-span performance and supervises the clustering assignment in a unified fashion. Clusters are determined by both covariate data (***X*** and the response vector (**Y**). In this manner, the approach allows us to take potential multicollinearity among exploratory variables into account, which would be very difficult to capture in a standard regression framework.

In this study, *Y*_*i*_, *i* = *1*, *…*, *N*, denotes the total number of words remembered for each individual and ${{\bf{X}}}_{i}=({X}_{i1}\ldots .,{X}_{ip})$ represents the covariate profile. Data for the outcome *Y*_*i*_ and the predictors **X**_*i*_ are modelled jointly, resulting in an infinite mixture model^44,45^:

1 $$f({Y}_{i},{{\boldsymbol{X}}}_{{\boldsymbol{i}}}|{\rm{\Theta }})=\sum _{c=1}^{\infty }{\psi }_{c}\,{P}_{r}({{\boldsymbol{X}}}_{{\boldsymbol{i}}}|{{\boldsymbol{\Theta }}}_{{\boldsymbol{c}}},{{\boldsymbol{\Theta }}}_{0})\,{P}_{r}({Y}_{i}|{{\boldsymbol{\Theta }}}_{{\boldsymbol{c}}},{{\boldsymbol{\Theta }}}_{0})$$

For each mixture component, the probability models for the outcome *Y*_*i*_ and the profile ***X***_***i***_ are independent conditionally on some specific parameters, ${{\boldsymbol{\Theta }}}_{{\boldsymbol{c}}}$, and some global parameter, ${{\boldsymbol{\Theta }}}_{0}$. The global parameters allow us to consider effects that potentially affect the response variable without being cluster specific (the so-called “fixed effects”). To facilitate inference, an additional allocation parameter *Z*_*i*_ was introduced so that *Z*_*i*_ = *c* indicates that individual *i* is assigned to cluster *c*. The mixture component weight is denoted by ${\psi }_{c}$ and represents the probability of subjects being assigned to the *c*^*th*^ cluster (${\psi }_{c}={P}_{r}({Z}_{i}=c)$) The mixture weights $\,{\boldsymbol{\psi }}=({{\rm{\psi }}}_{1,}\ldots ,{{\rm{\psi }}}_{{\rm{c}}}$) were modelled according to a stick-breaking prior^46,47^. The number of clusters was not fixed in advance but informed by the structure of data. In this respect, it is possible to approximate the infinite mixture model with a finite one by specifying a maximum number C of components^44^.

The probability model in Eq. (1) suggests that the methodology relies on two key components: the *profile sub-model* ${P}_{r}({{\bf{X}}}_{i}|{Z}_{i,}{{\boldsymbol{\Theta }}}_{zi},{{\boldsymbol{\Theta }}}_{0})$, which assigns individual profiles to clusters, and the *response sub-model* ${P}_{r}({{\bf{Y}}}_{i}|{Z}_{i,}{{\boldsymbol{\Theta }}}_{zi},{{\boldsymbol{\Theta }}}_{0})$ which links cluster of profiles to the outcome of interest via a regression model. In the original formulation, *the response sub-model* handles a binary outcome, common in epidemiological applications, but can be easily tailored to different response variables.

Here, we modelled affective O-span performance as a binomial variable. To perform the BPR analysis the total number of words remembered and the number of positive, negative and neutral words remembered were considered as outcome variables. For the *profile sub-model*, genotype, gender, age, education, general working memory, verbal fluency and mood were considered as covariates. The ordinal variables were dichotomized using median values as cut-off.

In the *profile sub-model*, for *p* covariates, with *M*_*p*_ the number of possible categories for covariate *p*, we assumed locally independent variables given the allocation variable *Z*_*i*_ = *c*, such that ${X}_{{i}_{p}}|{Z}_{i}=c$ ~ Multinomial $({\varphi }_{c}^{(p)})$, $p=1,\ldots ,P$^14^. Here, ${{\boldsymbol{\phi }}}_{{\boldsymbol{p}}}^{({\boldsymbol{c}})}=({\phi }_{p}^{(c)}(1),\ldots ,({\phi }_{p}^{(c)}({M}_{p}))$ and ${\phi }_{p}^{(c)}$(m) is the probability the *p*^*th*^ covariate in the cluster *c* is equal to *m*.

Owing to the complexity of the model, inference was set up within a Bayesian framework. Joint inference on the full set of parameters for such mixture models was facilitated by Markov Chain Monte Carlo (MCMC) methods^48^. At each iteration of the MCMC samplers, individual covariate profiles were first assigned to clusters and then affective O-span performance associated with a given group was assigned to each subject in the cluster^49–51^.

## Supplementary information

Supplementary Figures 1, 2, 3, 4

## Data Availability

All data generated or analyzed during this study are included in this published article (and its Supplementary Information files).

## References

[1] Park DC (2002). Models of visuospatial and verbal memory across the adult life span. Psychol. Aging.

[2] Salthouse TA (1996). The Processing-Speed Theory of Adult Age Differences in Cognition. Psychol. Rev..

[3] Craik, F. I. M. & Byrd, M. *In Aging and Cognitive Processes*, 10.1007/978-1-4684-4178-9_11 (1982).

[4] Hasher L, Zacks RT (1988). WorkingMemory, Comprehension, and Aging: A Review and a New View. Psychol. Learn. Motiv. - Adv. Res. Theory..

[5] Baddeley, A., Hitch, G. & (Ed.) G. H. B. Working Memory. *Psychol*. *Learn*. *Motiv*. **2**, 10.1016/j.cub.2009.12.014 (1974).

[6] Barrouillet P, Camos V (2014). On the proper reading of the TBRS model: Reply to Oberauer and Lewandowsky. Front. Psychol..

[7] Mather M (2016). The Affective Neuroscience of Aging. Annu Rev Psychol..

[8] Mather M, Carstensen LL (2005). Aging and motivated cognition: The positivity effect in attention and memory. Trends Cogn Sci..

[9] Carstensen LL, Mikels JA (2005). At the Intersection of Emotion and Cognition. Curr Dir Psychol Sci..

[10] Grühn D, Smith J, Baltes PB (2005). No aging bias favoring memory for positive material: Evidence from a heterogeneity-homogeneity list paradigm using emotionally toned words. Psychol Aging..

[11] Reed AE, Chan L, Mikels JA (2014). Meta-analysis of the age-related positivity effect: Age differences in preferences for positive over negative information. Psychol Aging..

[12] Satpute A, Lieberman M (2006). Integrating automatic and controlled processes into neurocognitive models of social cognition neurocognitive models of social cognition. Brain Res..

[13] Kalisch R (2006). Context-Dependent Human Extinction Memory Is Mediated by a Ventromedial Prefrontal and Hippocampal Network. J Neurosci..

[14] Mather M (2004). Amygdala Responses to Emotionally Valenced Stimuli in Older and Younger Adults. Psychol Sci..

[15] Wright CI, Wedig MM, Williams D, Rauch SL, Albert MS (2006). Novel fearful faces activate the amygdala in healthy young and elderly adults. Neurobiol Aging..

[16] Mammarella N (2016). The modulating role of ADRA2B in emotional working memory: Attending the negative but remembering the positive. Neurobiol Learn Mem..

[17] Balsamo M, Cataldi F, Carlucci L, Fairfield B (2018). Assessment of anxiety in older adults: A review of self-report measures. Clin Interv Aging..

[18] Fairfield B (2018). A variant on promoter of the cannabinoid receptor 1 gene (CNR1) moderates the effect of valence on working memory. Memory..

[19] Todd RM (2015). Neurogenetic variations in norepinephrine availability enhance perceptual vividness. J Neurosci..

[20] Rasch B (2009). A genetic variation of the noradrenergic system is related to differential amygdala activation during encoding of emotional memories. Proc Natl Acad Sci USA.

[21] Todd RM (2013). Genes for Emotion-Enhanced Remembering Are Linked to Enhanced Perceiving. Psychol Sci..

[22] Molnar S (2010). Comparative study on gene tags of the neurotransmission system in schizophrenic and suicidal subjects. Coll Antropol..

[23] Fairfield B (2017). The ADRA2B gene in the production of false memories for affective information in healthy female volunteers. Behav Brain Res..

[24] De Quervain DJF (2007). A deletion variant of the α2b-adrenoceptor is related to emotional memory in Europeans and Africans. Nat Neurosci..

[25] Li SJ, Weerda R, Guenzel F, Wolf OT, Thiel CM (2013). ADRA2B genotype modulates effects of acute psychosocial stress on emotional memory retrieval in healthy young men. Neurobiol Learn Mem..

[26] Todd RM (2014). Deletion variant in the ADRA2B gene increases coupling between emotional responses at encoding and later retrieval of emotional memories. Neurobiol Learn Mem..

[27] Zoladz PR (2014). ADRA2B deletion variant selectively predicts stress-induced enhancement of long-term memory in females. Psychoneuroendocrinology..

[28] Mammarella N, Di Domenico A, Palumbo R, Fairfield B (2016). Noradrenergic modulation of emotional memory in aging. Ageing Res Rev..

[29] Mather M, Harley CW (2016). The Locus Coeruleus: Essential for Maintaining Cognitive Function and the Aging Brain. Trends Cogn Sci..

[30] Alteba S, Korem N, Akirav I (2016). Cannabinoids reverse the effects of Early stress on neurocognitive performance in adulthood. Learn Mem..

[31] Mclaughlin RJ, Gobbi G (2012). Cannabinoids and Emotionality: a Neuroanatomical Perspective. Neuroscience..

[32] Li CR, Sinha R (2008). Inhibitory control and emotional stress regulation: Neuroimaging evidence for frontal – limbic dysfunction in psycho-stimulant addiction. Biobehav Rev..

[33] Loureiro M, Renard J, Zunder J, Laviolette SR (2015). Hippocampal cannabinoid transmission modulates dopamine neuron activity: impact on rewarding memory formation and social interaction. Neuropsychopharmacology..

[34] Moreira FA, Lutz B (2008). The endocannabinoid system: emotion, learning and addiction. Addict Biol..

[35] Morena M, Campolongo P (2014). The endocannabinoid system: An emotional buffer in the modulation of memory function. Neurobiol Learn Mem..

[36] Tan H, Ahmad T, Loureiro M, Zunder J, Laviolette SR (2014). The Role of Cannabinoid Transmission in Emotional Memory Formation: Implications for Addiction and Schizophrenia. Front Psychiatry..

[37] Beck DM, Kastner S (2009). Top-down and bottom-up mechanisms in biasing competition in the human brain. Vision Res..

[38] Robertson IH (2013). A noradrenergic theory of cognitive reserve: implications for Alzheimer’s disease. Neurobiol Aging..

[39] Turner ML, Engle RW (1989). Is working memory capacity task dependent?. Journal of Memory and Language..

[40] Montefinese M, Ambrosini E, Fairfield B, Mammarella N (2014). The adaptation of the Affective Norms for English Words (ANEW) for Italian. Behav Res Methods..

[41] Koutroumani M (2013). The deletion variant of alpha2b-adrenergic receptor is associated with decreased risk in Alzheimer’s disease and mild cognitive impairment. J Neurol Sci..

[42] Wechsler, D. Manual for the Wechsler Adult Intelligence Scale - Revised, Psychological Corporation (1981).

[43] Watson D, Clark LA, Tellegen A (1988). Development and Validation of Brief Measures of Positive and Negative Affect: The PANAS Scales. J Pers Soc Psychol..

[44] Molitor J, Papathomas M, Jerrett M, Richardson S (2010). Bayesian profile regression with an application to the National survey of children’ s health. Biostatistics..

[45] Hastie DI, Liverani S, Azizi L, Richardson S, Stücker I (2013). 2013 A semi-parametric approach to estimate risk functions associated with multi-dimensional exposure profiles: application to smoking and lung cancer. BMC Med Res Methodol..

[46] Ishwaran H, James LF (2001). Gibbs Sampling Methods for Stick Breaking Priors. J Am Stat Assoc..

[47] Sethuraman J (1994). A constructive definition of Dirichlet Priors. Stat Sin..

[48] Gilks, W., Richardson, S., Spiegelhalter, D. Markov chain Monte Carlo in practice (1995).

[49] Mammarella N (2012). Is there an affective working memory deficit in patients with chronic schizophrenia?. Schizophr Res..

[50] Mammarella N, Borella E, Carretti B, Leonardi G, Fairfield B (2013). Examining an emotion enhancement effect in working memory: Evidence from age-related differences. Neuropsychol Rehabil..

[51] Liverani S, Hastie DI, Azizi L, Papathomas M, Richardson S (2013). PReMiuM: An R Package for Profile Regression Mixture Models using Dirichlet Processes. J Stat Softw..

