# Supplementary material for: Aging and the Combined effects of ADRA2B and CB1 deletions on Affective Working Memory
Source: Sci Rep. 2019 Mar 11;9:4081. doi: 10.1038/s41598-019-40108-5 (PMC6411975; doi:10.1038/s41598-019-40108-5)

**Aging and the Combined effects of ADRA2B and CB1 deletions on Affective Working Memory**

Beth Fairfield ^1,2, *^, Nicola Mammarella ^1,2^, Lara Fontanella^3^, Annalina Sarra^3^, Marco D’Aurora^1,2^, Liborio Stuppia^1,2^, Valentina Gatta ^1,2^

^1^ Department of Psychological, Health and Territorial Sciences, ”G. d’Annunzio” University, Chieti-Pescara, Via Dei Vestini 31, 66100, Chieti, Italy

^2^ CeSI-Met, ”G. d’Annunzio” University, Chieti-Pescara, Via Dei Vestini 31, 66100, Chieti, Italy

^3^ Department of Economics, ”G. d’Annunzio” University, Chieti-Pescara, Viale Pindaro 42, 65127, Pescara, Italy

^*^ Beth Fairfield should be regarded as the corresponding author. Department of Psychological, Health and Territorial Sciences, School of Medicine and Health Sciences, “G.d’Annunzio” University, Via Dei Vestini 31, 66100, Chieti, Italy. Phone: (39) 08713554167; Fax: (39) 0871541300; Email: [bfairfield@unich.it](mailto:bfairfield@unich.it)

Supp. Figure 1

Number of positive words remembered as response variable: summary plot of the posterior distribution of parameter $\boldsymbol{\phi}_{c}$ , for *c* = 1,…, 6.


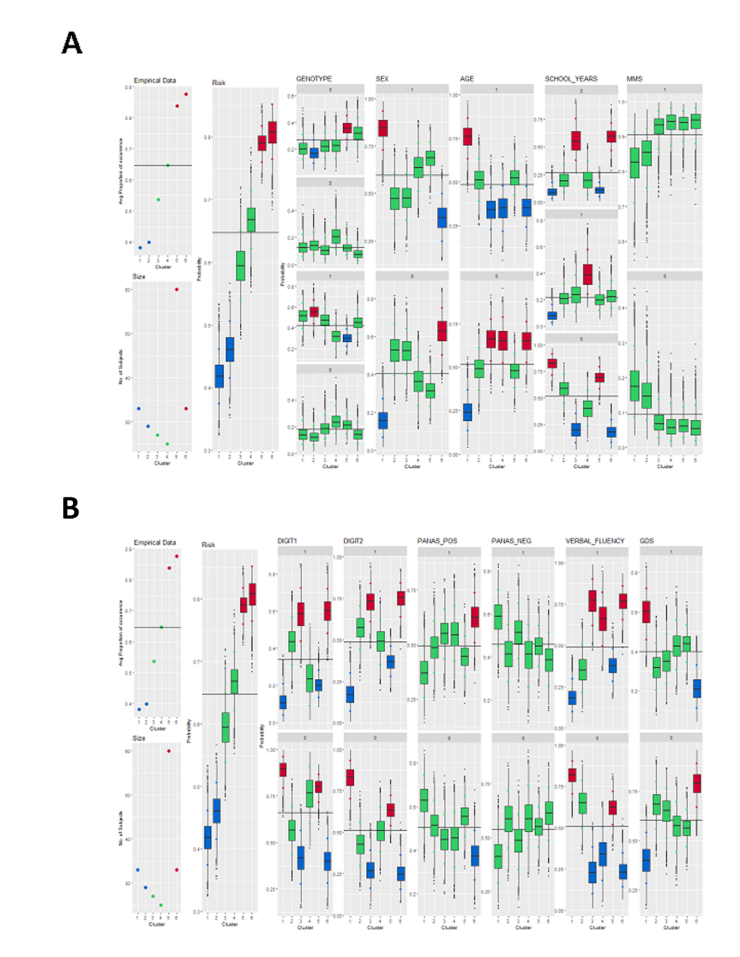


Supp. Figure 2

Number of negative words remembered as response variable: summary plot of the posterior distribution of parameter $\boldsymbol{\phi}_{c}$ , for *c* = 1,…, 5.


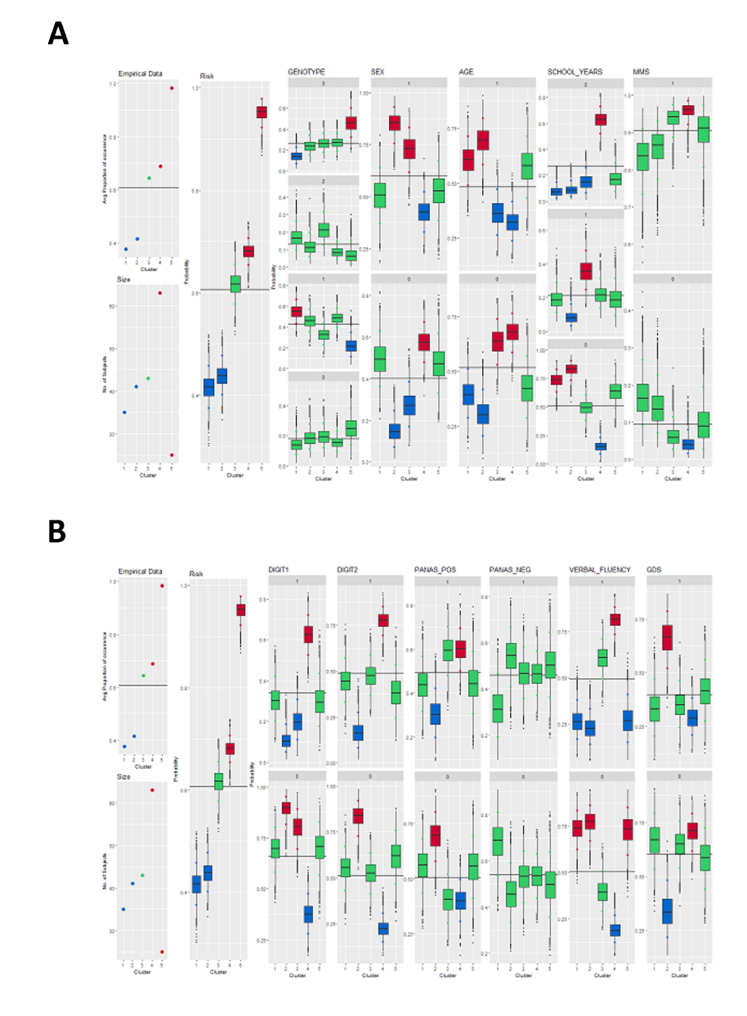


Supp. Figure 3

Number of neutral words remembered as response variable: summary plot of the posterior distribution of parameter $\boldsymbol{\phi}_{c}$ , for *c* = 1,…, 6.


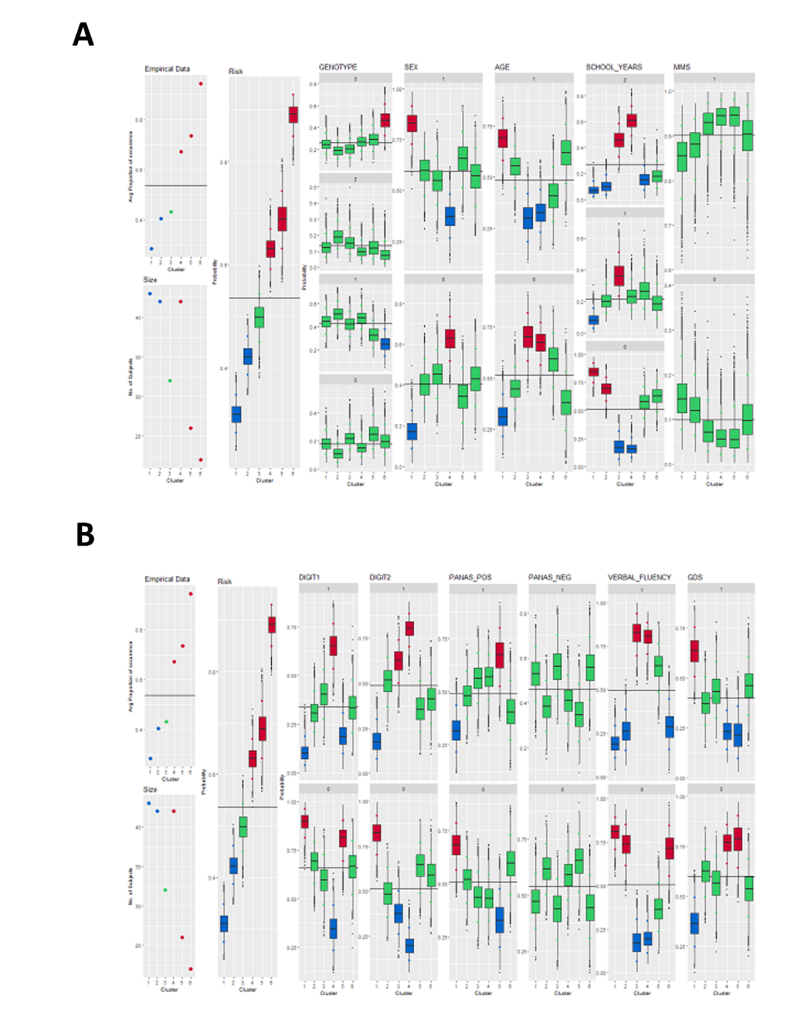


Supp. Figure 4

Heatmap of genotype distribution and O-span test achievements. In the upper panel of Figure 4, the white colour indicates the presence of gene variants while the black the absence. In the other panels, the white colour designates higher percentages of words remembered and best groups, respectively.


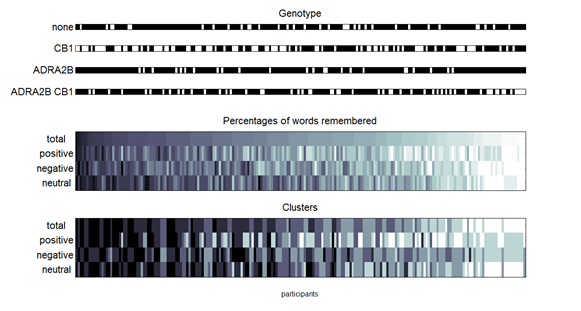

Supplement: Supplementary file 1 — Supplementary Figures 1, 2, 3, 4 [file 41598_2019_40108_MOESM1_ESM.docx]
